# Efficacy and safety of a two-step method of skin preparation for peripheral intravenous catheter insertion: a prospective multi-centre randomised trial

**DOI:** 10.1186/1471-2253-7-1

**Published:** 2007-01-31

**Authors:** Nathalie L van der Mee-Marquet, the Bloodstream infection Study Group of the Relais Régional d'Hygiène Hospitalière du Centre

**Affiliations:** 1 Service de Bactériologie et d'Hygiène, Hôpital Trousseau, Centre Hospitalier Universitaire, 37044 Tours, France

## Abstract

We have developed a two-step procedure for preparing the skin before peripheral venous catheter (PVC) insertions. This procedure involves two successive swabbings with wipes soaked in alcoholic antiseptic. We investigated whether this two-step procedure was as effective and safe as the standard four-step procedure – washing with detergent, rinsing, drying, applying antiseptic – by carrying out a multicentre randomised equivalence study comparing the frequency of precursor signs of infection at the site of insertion for the two skin preparation procedures. The study was carried out over an eight-month period, and 248 PVC insertion sites were evaluated. The two-step procedure was used for 130 subjects and the standard procedure for 118. Taking into account all the confounding factors predisposing patients to the complications studied, the characteristics of the two groups of patients were found to be similar, with no significant differences noted. The incidence of precursor signs of infection was 11 % 24 hours after PVC insertion (27/248), 25 % at 48 hours (50/203) and at 29 % at 72 hours (34/119). Eleven patients had complications necessitating the withdrawal of the PVC: sensitivity of the insertion site, with redness and/or slight swelling and/or a palpable venous cord. No major complications were observed in this study. The frequency of local complications associated with PVCs reported in this study, whether simple or severe, was not affected by the skin preparation procedure used for PVC insertion (two-step or four-step procedure).

## Background

Peripheral venous catheters (PVCs) are frequently used in hospitalised patients. The insertion of a PVC is an invasive act that may lead to local complications, thrombophlebitis, and infection of the PVC, which may cause bacteraemia or fungaemia [1-3]. Bacteraemia associated with the use of PVCs is rare (<0.1 to 0.2%) [1], but the high frequency of PVC use nonetheless results in high morbidity levels. The risk factors for complications associated with the use of PVCs relate to the patient (age, sex, associated disease, nearby infectious focus), the catheter (type of material, size), its insertion and the care administered (site of insertion, experience of the healthcare worker, lack of asepsis, duration of catheterisation, number of interventions involving the PVC, quality of maintenance of the device) and the solutes perfused (composition, pH, osmolarity, infusion rate) [2-8]. Most of the complications associated with the use of PVCs are avoidable [9]. Their prevention is based on good hand hygiene before insertion and during maintenance, choice of insertion site, the use of aseptic techniques for insertion and manipulation of the catheter, catheter attachment and limitation of the use of hypo- and hypertonic solutions [1,10-15].

We have developed a two-step procedure for skin preparation before the insertion of PVCs, involving two successive swabbings with wipes soaked with alcoholic antiseptic. We carried out a multicentre study comparing this two-step procedure with a standard four-step procedure – washing with detergent, rinsing, drying, application of antiseptic – to determine whether the two procedures were equally effective and safe.

## Materials and methods

### Design of study

The *Relais Régional d'Hygiène Hospitalière du Centre *(RHC), the centre responsible for monitoring nosocomial infections in the Centre Region of France (2.5 million inhabitants), aims to decrease the risk of infection associated with the use of intravenous devices. In 2004, we asked healthcare centres of the OUEST inter-region of France to participate in a multi-centre equivalence study, stratified by centre and comparing, in parallel, two skin preparation procedures for PVC insertion: a two-step procedure (A) and the standard four-step procedure (B). The study was co-ordinated by the RHC, the staff of which wrote the study protocol, trained the investigators, prepared randomisation lists entered and analysed data from the observation notebooks. Ethical approval of the trial was obtained from the Scientific Council of the CCLIN Ouest and Current Controlled Trials has assigned the following ISRCTN to the trial : ISRCTN01075518.

Twelve healthcare institutions (11 hospitals and clinics, one long-term care centre) participated in this study, which was carried out between February 1^st ^and August 31^st ^2004. In each participating centre, two groups of patients were selected, randomised and stratified by centre (group A treated with procedure A, group B with procedure B). The investigators informed the heads of nursing care in their institutions, informed and trained health managers and nurses, centralised the randomisation list, attributed each patient to a skin preparation procedure group on inclusion, monitored the running of the study and collected the results for their centres.

### Inclusion of patients

The investigators were responsible for including patients in the study. This involved asking eligible patients to participate, providing information about the protocol, presenting the letter of information and the consent form and collecting the consent form after the patient had been allowed time to consider their participation, preparing an observation notebook, randomising skin preparation protocols and noting the prescription on the data collection form, with randomisation. The inclusion criteria were as follows: hospitalised, consenting adults aged over 18 years, male or female, requiring the insertion of a continuous PVC with a treatment duration of more than 48 hours, and those for whom either skin preparation procedure could be used. The non-inclusion criteria were medical reasons, the insertion of a catheter for emergency perfusion, allergy to povidone, a planned intravenous treatment time of less than 48 h, the presence of skin lesions at the chosen insertion site, being in the last three months of pregnancy, refusal to give consent, patient unconscious or incapable of understanding the information given. The number of subjects required for this equivalence study was estimated at 231 subjects per group, assuming a frequency of pre-infection signs of 25 to 30%, and a difference of 5% between the frequencies in the two groups for equivalence of the two techniques of skin preparation and a study power of 80% (Nquery software).

### The two procedures of skin preparation

Procedures A and B are described in table 1. Povidone iodine is a first-line antiseptic with prolonged activity and alcohol is a fast-acting antiseptic routinely used to disinfect injection sites. The use of an antiseptic in alcoholic solution results in rapid, strong and persistent bactericidal activity, with more rapid drying than for aqueous solutions. Povidone iodine in ethanol (Bétadine^®^, 5% povidone iodine in ethanol), which is used for skin disinfection before surgery [16], was chosen for this procedure. Procedure B involved the use of a detergent solution (Bétadine Scrub^®^, povidone iodine), sterile water (injectable, 10 ml ampoule) and an antiseptic (Bétadine Dermique^®^, 10% povidone iodine). The choice of product was based on authorisation for market release for PVC insertion, knowledge of application times and packaging in 10 ml single-use containers, facilitating their use in this study. The RHC provided the participating institutions with these products and the departments within the institutions were supplied with the products by their pharmacists.

**Table 1:** Details of the two-step procedure (A) and the standard four-step procedure (B)

	Two-step procedure (A)	Standard four-step procedure (B)
**Products**	Alcoholic antiseptic solution [Bétadine^® ^5 % in alcohol, iodine antiseptic]	Detergent solution [Bétadine^® ^Scrub, povidone iodine] Sterile water [injectable, 10 ml ampoule] Antiseptic solution [Bétadine^® ^Dermique 10% in alcohol, povidone-iodine]
**Procedure**	Extensive application of the alcoholic antiseptic with sterile wipes. Skin left to dry in air for 30 seconds Second application of alcoholic antiseptic with a fresh sterile wipe. Skin left to dry in air for 30 seconds before insertion of the PVC	Application of the foaming detergent solution. Rinsing, with sterile compresses imbibed with sterile water. Drying with sterile compresses. Application of antiseptic, using sterile compresses imbibed with the product. Skin left to dry in air for 1 minute before insertion of the PVC.

### Insertion of catheters

PVCs were inserted and maintained by nursing staff. The investigators were responsible for checking that the prescribed skin preparation protocol was adhered to and for collecting data concerning possible confounding factors. These included the age of the patient, immunosuppression, nature of the catheter, number of manipulations per day, duration of catheterisation and the nature of the perfused solutes.

### Evaluation of the appearance of precursor signs of infection at the insertion site

The ideal way to determine whether the two-step procedure was as effective and safe as the standard four-step procedure would have been to compare the incidence of infections linked to PVCs as a function of skin preparation procedure. However, this would have required a much larger series of patients, which would be difficult to achieve nowadays in a context of medical wards with overworked staff. Observations of precursor signs of infections associated with PVCs, and comparisons of their frequency as a function of certain criteria (nature of the catheter, antiseptic used, insertion site, etc.) have been used to study the risk factors for PVC-linked infections [4,14,17]. We therefore carried out a randomised equivalence study, comparing the frequency of precursor signs of infection at the site of insertion for the two skin preparation procedures.

Inspection of the insertion site is a key part of nursing care. Patients were monitored daily throughout the time of perfusion, until withdrawal of the catheter, for a maximum of 72 h. The judgement criterion was the appearance of precursor signs of infection at the insertion site, as evaluated according to the Maddox scale (detailed in table 2) [17]. The nurses were trained in the use of this scale before the start of the study. The indications for catheter removal were evaluated each day. Catheters that were no longer required were removed. Catheters were also systematically removed if major precursor signs of infection were observed.

**Table 2:** The Maddox scale

Index	Complications	Clinical signs
0	None	No signs
1	Simple	Sensitivity or redness at the insertion site
2	Simple	Sensitivity of the insertion site, with redness or slight swelling
3	Severe	Sensitivity of the insertion site, with redness and slight swelling or a palpable venous cord
4	Severe	Sensitivity of the insertion site, with redness, slight swelling and a palpable venous cord
5	Major	All the signs listed for index 4 plus purulence

### Data analysis

We began by describing the study sample, to check that groups A and B were comparable in terms of confounding factors. The principal analysis then involved comparing the frequency of precursor signs of infection in the two groups. The judgement criterion was a qualitative, six-class (0–5) variable for each group of subjects. We therefore calculated the percentage and confidence interval for each class and compared the percentages between groups by means of a χ^2 ^test with five degrees of freedom. The secondary analyses, with adjustment for potential confounding factors, were then carried out.

## Results

The study was initially planned to run for six months and required meticulous, fastidious and committed work from the investigators, particularly as it was necessary to seek the patients' consent. Despite a two-month prolongation, and due to the difficulties encountered in trying to recruit patients, the planned number of patients was not reach and the final analysis was based on only 248 files.

### Insertion of catheters

We evaluated 248 PVC insertion sites for early signs of infection. The number of cases included in each centre varied from 2 to 54 (median value 14). Procedure A was carried out for 130 subjects and procedure B, for 118. The characteristics of the patients and the confounding factors are presented in table 3. Taking into account all the confounding factors predisposing patients to the complications studied, the characteristics of the two groups of patients were found to be similar, with no significant differences noted.

**Table 3:** Characteristics of the patients of groups A and B as a function of confounding factors

	Number of patients			p*
		
	Total	By procedure		
		A	B	
Number of PVCs	248	130	118	
Sex ratio (M/F)	155/93	84/46	71/47	0.47
Age > 70 years	88	42	46	0.27
Immunosuppression	62	32	30	0.88
Insertion site				
Back of the hand	35	20	15	0.91
Elbow	17	8	9	
Forearm	190	99	91	
Other	6	3	3	
Nature of the PVC				
Polyurethane	150	79	71	0.79
Polyvinyl chloride	57	28	29	
Teflon	41	23	18	
Duration of catheterisation				
> 24 h and < 48 h	248	130	118	0.87
> 48 h and < 72 h	203	108	95	
72 h	119	63	56	
Mean duration	> 48 h and < 72 h	> 48 h and < 72 h	> 48 h and < 72 h	
No. manipulations > 4/day	52	29	23	0.58
Solutes perfused				
Antibiotics	85	45	40	0.90
Anticancer drugs	57	30	27	0.97
Lipids	9	5	4	0.85
Blood products	7	5	2	0.31

*Chi squared was calculated with values obtained for groups A and B.

### Incidence of complications and association with the skin preparation procedure

In total, 811 insertion site inspections were carried out: 45 PVCs were evaluated on a single occasion, because they were removed within 48 hours, 84 PVCs were evaluated on two occasions and were removed within 72 hours, and the remaining 119 PVCs were evaluated on three occasions and removed after 72 hours in place. The incidence of complications in the two groups, as evaluated by the Maddox index [17], is shown in table 4. The results are expressed as a function of duration of catheterisation, with three groups, corresponding to patients with PVCs in place for up to 24 hours, 48 hours and 72 hours. The overall incidence of precursor signs of infection was low in both groups for a catheterisation for up to 24 hours, with 89 % (221/248) of patients having no complications (Maddox index = 0). Complications were observed in 50 of the 203 (25 %) patients with PVCs in place for up to 48 hours, and in 34 of the 119 (29 %) patients with PVCs in place for 72 hours. Eleven patients had severe complications (Maddox index 3 and 4). The PCV was withdrawn in each of these 11 cases. The largest number of severe complications (4 of 63) occurred in patients with procedure A skin preparation and PVCs left in place for 72 hours. However, the actual numbers were so small that they did not differ significantly from those for group B. No major complications (Maddox index 5) were observed in this study. The frequency of complications, whether simple or severe, did not differ significantly between groups A and B.

**Table 4:** Early signs of infection, evaluated by the Maddox index, as a function of the skin preparation procedure used

	No. of insertion site observations (%)					
	24 hours after insertion		48 hours after insertion		72 hours after insertion	
	By procedure					
Maddox index	A (n = 130)	B (n = 116)	A (n = 109)	B (n = 94)	A (n = 63)	B (n = 56)
0	112 (86%)	107 (92%)	81 (74%)	72 (77%)	44 (70%)	41 (73%)
1 Simple	12	7	19	12	11	12
2 Simple	5	3	7	8	4	2
Total – simple	17 (13%)	10 (9%)	26 (24%)	20 (21%)	15 (24%)	14 (25%)
3 Severe	0	1	2	1	1	0
4 Severe	1	0	0	1	3	1
Total – severe	1 (<1%)	1(<1%)	2 (2%)	2 (2%)	4 (6%)	1 (2%)
5 Major	0	0	0	0	0	0

Number of PVCs with precursor signs of infections	18 (14%)	9 (8%)	28 (26%)	22 (23%)	19 (30%)	15 (27%)
	27 (11%)		50 (25%)		34 (29%)	
p*	0.127		0.706		0.684	

*Chi squared was calculated for numbers of PVCs displaying precursor signs of infections in groups A and B.

### Frequency of complications and risk factors

The precursor signs of infections in both groups (A and B) associated with the various known risk factors are listed in table 5. For all studied risk factors, no significant differences could be seen between the groups.

**Table 5:** Early signs of infection according to the skin preparation procedure used and the known risk factors. Number of patients (%)

48 hours after insertion	Maddox index 0		Maddox index 1–2		Maddox index 3–5	
	A	B	A	B	A	B
PVCs	81	72	26	20	2	2
Men	51 (63)	46 (64)	17 (65)	11 (55)	2 (100)	2 (100)
Age > 69 years	28 (34)	28 (39)	8 (31)	8 (40)	1 (50)	1 (50)
Immunosuppression	20 (25)	20 (28)	5 (19)	5 (25)	-	-
Insertion site						
Back of the hand	14 (17)	9 (12)	3 (11)	1 (5)	-	-
Elbow	5 (6)	5 (7)	1 (4)	2 (10)	-	-
Forearm	62 (76)	55 (76)	20 (77)	17 (85)	2 (100)	2 (100)
Nature of PVC						
Polyurethane	52 (64)	44 (61)	11 (42)	12 (60)	2 (100)	1 (50)
Polyvinyl chloride	13 (16)	8 (11)	9 (35)	4 (20)	-	1 (50)
Teflon	18 (22)	20 (28)	4 (15)	4 (20)	-	-
Solutes perfused						
Antibiotics	31 (38)	26 (36)	9 (35)	7 (35)	-	2 (100)
Anticancer drugs	20 (25)	18 (25)	4 (15)	4 (20)	-	-
Lipids	2 (2)	-	-	2 (10)	-	-
Blood products	3 (4)	-	1 (4)	1 (5)	-	-

72 hours after insertion	Maddox index 0		Maddox index 1–2		Maddox index 3–5	
	A	B	A	B	A	B

PVCs	44	41	15	14	4	1
Men	30 (68)	28 (68)	8 (53)	8 (57)	3 (75)	1 (100)
Age > 69 years	17 (39)	15 (37)	7 (47)	4 (29)	1 (25)	-
Immunosuppression	12 (27)	12 (29)	5 (33)	6 (43)	-	12 (27)
Insertion site						
Back of the hand	6 (14)	4 (10)	4 (27)	-	1 (25)	-
Elbow	3 (7)	3 (7)	1 (7)	1 (7)	-	-
Forearm	34 (77)	32 (78)	9 (60)	13 (93)	3 (75)	1 (100)
Nature of PVC						
Polyurethane	26 (59)	23 (56)	5 (33)	5 (36)	1 (25)	1 (100)
Polyvinyl chloride	8 (18)	5 (12)	4 (27)	3 (21)	3 (75)	-
Teflon	11 (25)	13 (32)	5 (33)	6 (43)	-	-
Solutes perfused						
Antibiotics	20 (45)	13 (32)	5 (33)	3 (21)	3 (75)	-
Anticancer drugs	12 (27)	12 (29)	5 (33)	5 (36)	-	-
Lipids	-	-	1 (7)	1 (7)	-	-
Blood products	-	-	2 (13)	-	-	-

### Skin tolerance and alcoholic antiseptics

Alcoholic antiseptics have been reported to cause chemical burns in some cases [18]. The investigators were asked to note any abnormalities. No skin reaction was reported during the study in patients from either of the two groups.

## Discussion

The insertion site of the catheter is the principal source of PVC contamination, and the density of the skin microflora at the insertion site is a major risk factor for PVC-linked bacteraemia [3,8,19]. Strict compliance with skin preparation procedures before insertion of the PVC is one of the principal means of preventing PVC-linked infection [20]. The American Centers for Disease Control (CDC) recommend the disinfection of clean skin with an appropriate antiseptic, using a sufficiently long contact time and allowing the skin to dry in the air before inserting the catheter [15]. The French guidelines recommend the use of two products (a detergent and an antiseptic) in four steps (washing with detergent, rinsing, drying, application of antiseptic) for skin preparation [21].

A lack of training, underestimation of the risk of complications associated with the use of PVCs and a too-heavy workload often result in poor adherence with procedures for PVC insertion in routine practice [10,11,22,23]. In 2002, an audit of skin preparation before the insertion of PVCs carried out in six healthcare institutions in the Centre region of France showed a large gulf between theory and practice, with compliance with the recommended skin preparation techniques observed in only 30% of the 105 observations. This lack of compliance was attributed to the time-consuming nature of the recommended procedure. We therefore developed a two-step skin preparation procedure, with the aim of increasing compliance with disinfection procedures by simplifying the technique.

The two-step procedure involves two successive swabbings with wipes soaked in alcoholic antiseptic. As alcohol is an efficient solvent, the first swabbing replaces washing with detergent. The rapid biocidal activity of the alcohol, together with that of the antiseptic, starts to decrease the bacterial load immediately. As the alcoholic antiseptic, unlike detergent, does not generate an emulsion of organic matter, rinsing is unnecessary, and the volatility of the alcohol facilitates the rapid drying of the skin without the need for wiping. The second swabbing corresponds to the true antiseptic step.

A large number of patients would have been required to demonstrate equivalent efficacy and safety of the two methods by comparing the incidence of PVC-linked infections as a function of skin preparation procedure, because the incidence of bloodstream infections related to PVCs is low. Thrombophlebitis is a precursor sign of PVC infection and has been reported to occur after 24 hours in 12 to 34% of cases and after 48 hours in 36 to 65% of cases [6,11,16,17]. The observation of precursor signs of infection associated with PVCs, and the comparison of their frequency as a function of certain criteria has been used to evaluate risk factors for PVC-linked infections in several previous studies. A prospective double-blind study of 195 men evaluated the effect of inline intravenous filters on post-infusion thrombophlebitis and the bacterial colonisation of catheters [24]. A comparative study of different skin preparation methods for PVCs was conducted in 60 patients, with monitoring of the incidence of precursor signs of infection as a function of skin preparation methods [14]. Another study compared peripheral intravenous Teflon^® ^and Vialon^® ^catheters [4], assessing the incidence of thrombophlebitis for 170 PVCs. Based on these data, we carried out our randomised equivalence study comparing the frequency of precursor signs of infection at the site of insertion for the two procedures: the two-step procedure and the standard four-step procedure.

The 111 complications reported – 100 simple and 11 severe – highlight the necessity of strict indications for the use of PVCs, to limit the occurrence of incidents and accidents caused by the use of these devices. The frequency of precursor signs of infection associated with PVCs in our study was lower than that reported in previous studies [17], probably because of the care taken to ensure that skin preparation procedures were followed during the study. No bacteraemic infections were observed for any of the 248 PVCs, probably as a consequence of the small sample size and the immediate withdrawal of the 11 PVCs for which severe complications were observed.

In conclusion, the frequency of precursor signs of infection associated with PVCs reported in this study was not affected by the skin preparation procedure used for PVC insertion (two-step or four-step procedure). Despite the limitations of our study (due to fewer patients than intended being recruited), the two-step procedure appears to be as safe and effect a method of skin preparation for PVC insertion as the standard four-step method.

## Authors' contributions

NVDM had the initial idea for the study, participated in its design and co-ordination and wrote the manuscript

The members of the Bloodstream Infection Study Group of the Relais Régional d'Hygiène Hospitalière du Centre : P Amirault (Centre Hospitalier, 18100 Vierzon), P Besnard (Centre Hospitalier, 28401 Nogent le Rotrou), D Bloc (Service de Bactériologie et d'Hygiène, Hôpital Trousseau, Centre Hospitalier Universitaire, 37044 Tours), B Branger (CCLIN Ouest, 35033 Rennes), MF Boucher (Centre Hospitalier, 28205 Châteaudun), M Chabaud-Mayer (Centre Hospitalier Louis Pasteur, 28018 Chartres), C Chapon (Centre Hospitalier, 36019 Châteauroux), F Coulomb (Centre Hospitalier, 28102 Dreux), D Dansou (Centre Hospitalier, 28205 Châteaudun), M Decesvre-Fricheteaux (Service de Maladies Infectieuses, Centre Hospitalier Universitaire, 37044 Tours), C Decreux (Centre Hospitalier, 36019 Châteauroux), K El Haichouni (Centre Hospitalier, 29270 Carhaix), PL Etienne (Clinique Armoricaine de Radiologie, 22015 Saint Brieuc), C Fièvre (Centre Hospitalier, 36300 Le Blanc), F Fongauffier (Centre Hospitalier, 28205 Châteaudun), N Girard (Relais Régional d'Hygiène Hospitalière du Centre, 37044 Tours), Y. Guimard (Centre Hospitalier, 18016 Bourges), A Hamel (Centre de Convalescence, 44160 Pontchâteau), G Heduit (Centre de Convalescence, 44160 Pontchâteau), A Janin (Relais Régional d'Hygiène Hospitalière du Centre, 37044 Tours), O Lehiani (Centre Hospitalier, 18016 Bourges), MF Lhuillier (Centre Hospitalier, 18100 Vierzon), R Mauricette (Centre Hospitalier, 28102 Dreux), P Michaud (Centre Hospitalier, 18016 Bourges), O Milan (Centre Hospitalier Louis Pasteur, 28018 Chartres), C Mourens (Relais Régional d'Hygiène Hospitalière du Centre, 37044 Tours), C Neveu (Centre Hospitalier, 28102 Dreux), G Petit (Centre Hospitalier, 28401 Nogent le Rotrou), C Poilve (Centre de Convalescence, 44160 Pontchâteau), R Quentin (Service de Bactériologie et d'Hygiène, Hôpital Trousseau, Centre Hospitalier Universitaire, 37044 Tours), O Raffy (Centre Hospitalier Louis Pasteur, 28018 Chartres), D Ratovohery (Centre Hospitalier, 36019 Châteauroux), R Salame (Centre Hospitalier, 28401 Nogent le Rotrou), ML Sparfel (Centre Hospitalier, 29270 Carhaix), A Sylvestre (Centre Hospitalier, 36300 Le Blanc), I Voyer (Service de Bactériologie et d'Hygiène, Hôpital Trousseau, Centre Hospitalier Universitaire, 37044 Tours). NG, BB, FC, OL participated in the design of the study. All members made substantial contributions to data acquisition. NG performed the statistical analysis. RQ reviewed the intellectual content of the manuscript.

This work was supported by the *Centre de Coordination de la Lutte contre les Infections Nosocomiales Ouest (CCLIN Ouest)*, l'*Agence Régionale de l'Hospitalisation du Centre *and Tours University Hospital. We thank all the investigators and the staff of the operational hygiene teams and the departments of the participating healthcare establishments who made this study possible.

## Pre-publication history

The pre-publication history for this paper can be accessed here:



## References

[B1] Widmer AF (1996). Intravenous-related infections. Prevention and control of nosocomial infections.

[B2] Maki DG (1992). Infections due to infusion therapy. Hospital infections.

[B3] RAAD I (1998). Intravascular catheter-related infections. Lancet.

[B4] Jacquot C, Fauvage B, Bru JP, Croize J, Calop J (1989). Cathétérisme veineux périphérique: influence de la composition du cathéter dans l'apparition de thrombophébites. Ann Fr Anesth Réanim.

[B5] Kagel EM, Rayan GM (2004). Intravenous catheter complications in the hand and forearm. J Trauma.

[B6] Maki DG, Ringer M (1991). Risk factors for infusion-related phlebitis with small peripheral venous catheter. A randomized controlled trial. Ann Intern Med.

[B7] Macfarlane JT, Ward MJ, Banks DC, Pilkington R, Finch RG (1991). Risks from cannulae used to maintain intravenous access. Br Med J.

[B8] Mermel LA, McCormick RD, Springman SR, Maki DG (1991). The pathogenesis and epidemiology of catheter-related infection with pulmonary artery Swan-Ganz catheters: a prospective study utilizing molecular subtyping. Am J Med.

[B9] Harbarth S, Sax H, Gastmeier P (2003). The preventable proportion of nosocomial infections: an overview of published reports. J Hosp Infect.

[B10] Hirschmann H, Fux L, Podusel J, Schindler K, Kundi M, Rotter M, Wewalka G, EURIDIKI. European Interdisciplinary Committee for Infection Prophylaxis (2001). The influence of hand hygiene prior to insertion of peripheral venous catheters on the frequency of complications. J Hosp Infect.

[B11] Lundgren A, Wahren LK (1999). Effect of education on evidence-based care and handling of peripheral intravenous lines. J Clin Nurs.

[B12] Curran AT, Coia JE, Gilmour H, McNamee S, Hood J (2000). Multi-centre research surveillance project to reduce infections/phlebitis associated with peripheral vascular catheters. J Hosp Infect.

[B13] Couzigou C, Lamory J, Salmon-Ceron D, Figard J, Vidal-Trecan GM (2005). Short peripheral venous catheters: effect of evidence-based guidelines on insertion, maintenance and outcomes in a university hospital. J Hosp Infect.

[B14] Gabel KS, Geelhoed GW, Zalkind DL (1988). A comparative study of a new skin preparation method for peripheral intravenous lines. Am Surg.

[B15] Centers for Disease Control and Prevention (2002). Guidelines for the prevention of intravascular catheter-related infections. MMWR.

[B16] Arata T, Murakami T, Hirai Y (1993). Evaluation of povidone-iodine alcoholic solution for operative site disinfection. Postgrad Med J.

[B17] Tagalakis V, Kahn SR, Libman M, Blostein M (2002). The epidemiology of peripheral vein infusion thrombophlebitis: a critical review. Am J Med.

[B18] Liu FC, Liou JT, Hui YL, Hsu JC, Yang CY, Yu HP, Lui PW (2003). Chemical burn caused by povidone-iodine alcohol solution – a case report. Acta Anaesthesiol Sin.

[B19] Maki DG, Weise CE, Sarafin HW (1977). A semiquantitative culture method for identifying intravenous-catheter-related infection. N Engl J Med.

[B20] Dettenkofer M, Jonas D, Wiechmann C, Rossner R, Frank U, Zentner J, Daschner FD (2002). Effect of skin disinfection with octenidine dihydrochloride on insertion site colonization of intravascular catheters. Infection.

[B21] Société Française d'Hygiène Hospitalière. Haute Autorité de santé (2005). Prévention des Infections liées aux cathéters périphériques: Recommandations pour la pratique clinique.

[B22] Nelson RR, Tebbs SE, Richards N, Elliott TS (1996). An audit of peripheral catheter care in a teaching hospital. J Hosp Infect.

[B23] Mimoz O, Karim A, Mercat A, Cosseron M, Falissard B, Parker F, Richard C, Samii K, Nordmann P (1999). Chlorhexidine compared with povidone-iodine as skin preparation before blood culture. A randomized, controlled trial. Ann Intern Med.

[B24] Maddox RR, John JF, Brown LL, Smith CE (1983). Effect of inline filtration on postinfusion phlebitis. Clin Pharm.

